# Longitudinal NGAL and cystatin C plasma profiles present a high level of heterogeneity in a mixed ICU population

**DOI:** 10.1186/s12882-024-03477-2

**Published:** 2024-01-29

**Authors:** Daniela Jou-Valencia, Meint Volbeda, Jan G. Zijlstra, Jenny E Kootstra-Ros, Jill Moser, Matijs van Meurs, Jacqueline Koeze

**Affiliations:** 1 Department of Critical Care, University of Groningen, University Medical Center Groningen (UMCG), Hanzeplein 1, Groningen, 9713GZ The Netherlands; 2 Department of Pathology and Medical Biology, Medical Biology section, Laboratory for Endothelial Biomedicine & Vascular Drug Targeting Research, University of Groningen, UMCG, Groningen, The Netherlands; 3 Department of Laboratory Medicine, UMCG, Groningen, The Netherlands

**Keywords:** Cystatin C, NGAL, Heterogeneity, AKI, Timeline

## Abstract

**Background:**

NGAL and Cystatin C (CysC) as biomarkers for the early detection of AKI are subject to both pathophysiological, as well as patient related heterogeneity. The aim of this study was to investigate the timeline of plasma levels of NGAL and CysC during the first seven days of ICU admission in a mixed ICU population and to relate these to AKI severity during ICU stay. Via these means we aimed to bring clarity to the previously reported heterogeneity of these renal biomarkers.

**Methods:**

Prospective Observation Cohort. Consecutive patients admitted to adult ICU at an academic hospital in the Netherlands between 18-02-2014 and 31-03-2014 were included. Urine output, serum creatinine, plasma NGAL and CysC were recorded during the first seven days of ICU admission. Biomarker expression was analyzed based on KDIGO score and time of AKI diagnosis.

**Results:**

335 patients were included, 110 met KDIGO criteria for AKI. NGAL and CysC plasma levels were higher in AKI patients compared to non-AKI, high variability in individual values resulted in 56% of AKI patients having a false negative, and 32% of non-AKI patients having a false positive. Individual biomarker levels were variable, and no pattern based on KDIGO score was observed.

**Conclusions:**

Plasma NGAL and CysC as biomarkers for the early AKI detection may be subject to pathophysiological, and patient related heterogeneity. Further understanding of individual biomarker profiles may help in their application amongst mixed ICU populations.

**Trial Registration:**

The need for informed consent was waived by the Institutional Ethical Review Board of the University Medical Center Groningen (METc 2013 − 174) by Prof. dr. W.A. Kamps on May 17th 2013.

**Supplementary Information:**

The online version contains supplementary material available at 10.1186/s12882-024-03477-2.

## Introduction

Acute kidney injury (AKI) affects 29–81% of patients admitted to the intensive care unit (ICU) [1]. AKI is associated with short-, and long-term morbidity and mortality [2–4]. Despite extensive research on prevention and treatment of AKI, prognosis has not significantly improved over the last twenty-five years. This may be related to multiple factors including the complexity of underlying disease, patient heterogeneity, and late identification of AKI.

AKI is diagnosed using plasma creatinine (pCr) levels and/or a decrease in urine output (UO) [5]. These functional criteria become apparent once a critical point in renal injury has occurred, leading to loss of renal function. AKI diagnosis once damage has already occurred largely limits therapeutic options. This has stimulated the search for early, and more precise renal markers to identify AKI.

Various studies have described functional, damage, and pre-injury phase biomarkers which may improve early identification of AKI and the prediction of Major Adverse Kidney Events (MAKE) (death, need for renal replacement therapy (RRT), or worsened kidney function) [2–4, 6]. Two widely researched biomarkers are neutrophil gelatinase-associated lipocalin (NGAL) and Cystatin C (CysC).

NGAL is an iron-transporting protein which is expressed at low levels in many cell types including neutrophils and distal tubular cells [7]. Upon injury or infection, NGAL creates an iron-complex which may be renoprotective and bacteriostatic. Elevated NGAL levels can be detected as early as three hours following renal injury, peaking after 8–12 h and remain elevated up to five days after injury [3]. NGAL has proven to be predictive of AKI development and progression in ischemic and toxic kidney injury in different groups of patients [1, 3, 8].

CysC is a low molecular protein present in all cells, produced at a constant rate, filtered freely by the glomerulus, and almost completely reabsorbed and catabolized in the proximal tubules [2, 3]. Its expression reflects the ability of the kidneys to filtrate solutes. Increased plasma CysC has been shown to detect AKI 24–48 h earlier than pCr [9].

Early results in the use of AKI biomarkers in early identification of AKI in homogenous patient populations (e.g. post cardiac surgery) have been promising, however in larger studies with heterogenous populations these results could not be replicated. This may be related to the heterogeneity of both etiology and patient response to AKI, as well as timing of testing [10–12].

The aim of this study was to investigate the course of plasma levels of NGAL and CysC during the first seven days of Intensive Care Unit (ICU) admission in a mixed ICU population and to relate these to AKI severity during ICU stay. In this study we focus on two different types (damage and functional) of renal biomarker, their dynamics during the first seven days of ICU admission and their association. Hereby we aim to clarify the previously reported heterogeneity of these renal biomarkers.

## Materials and methods

### Study design

**Fig. 1:**
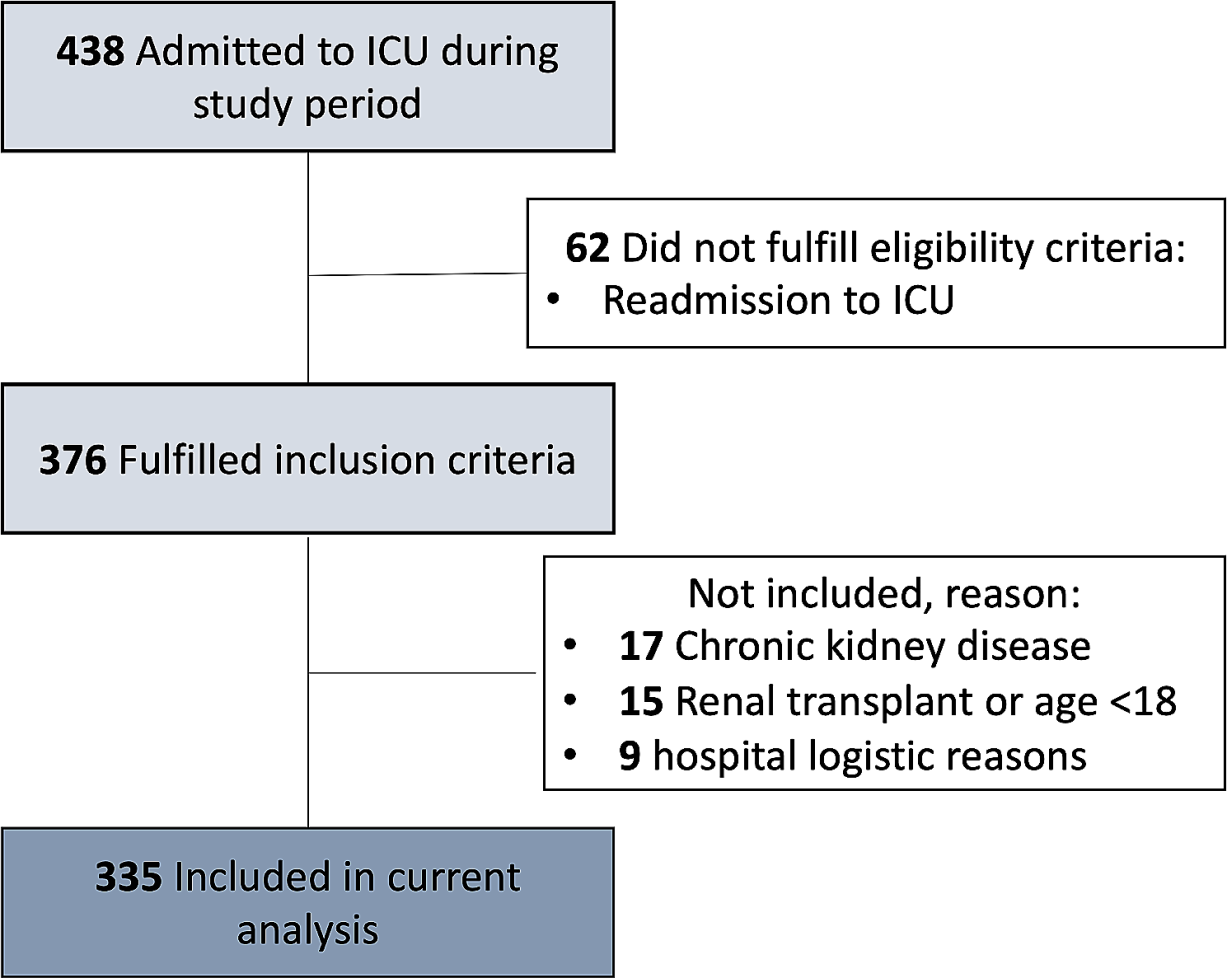
Overview patient inclusion

We conducted a prospective observational cohort study at a tertiary level academic hospital in the Netherlands. All patients admitted to the adult ICU between February 18th and March 31st, 2014 were included. Patient data were collected for the first consecutive seven days after ICU admission. In patients with multiple ICU admissions during the study period, only the first admission samples and data were used for the analysis. Patients under the age of 18, patients with chronic kidney disease (CKD) and renal transplant recipients were excluded from the study (Fig. 1). The study was performed in accordance with the Declaration of Helsinki. The need for informed consent was waived by the Institutional Ethical Review Board of the University Medical Center Groningen and all the experimental protocols were approved by " Institutional Ethical Review Board of the University Medical Center Groningen **“**(METc 2013 − 174). Detailed description of the study design has been previously described by Koeze et al. [13].

### Data collection

Hourly UO and daily pCr were recorded per patient. Routine plasma analyses were conducted upon admission, and routinely each day. Residual samples of plasma taken for clinical purposes were collected for separate biomarker analysis by the department of laboratory medicine. The results of the biomarker analysis were not reported in the patient data management system (PDMS), thereby blinding clinicians for the results. Moreover, all data were pseudo-anonymously entered into a database for further analysis. Due to limited number of patients with seven-day data, the first six days of admission were used in the data analysis.

The PDMS was retrospectively reviewed for possible cofounding factors and clinical outcomes.

### Plasma NGAL and CysC analysis by ELISA

NGAL was measured in routinely collected lithium heparin plasma samples using the BioPorto NGAL Test (Bioporto Diagnostics, Hellerup, Denmark) on a Roche Modular P chemistry platform (Roche, Mannheim, Germany). According to the manufacturer the NGAL test is validated for NGAL levels between 25 µg/L to 5000 µg/L. Overall coefficient of variation (CV) 2.9% at a level of 206 µg/L and 2.3% at a level of 511 µg/L.

CysC was measured in routinely collected lithium heparin plasma samples using the Gentian Cystatin C PETIA (particle enhanced turbidimetric immunoassay) reagents (standardized against ERM-DA 471, Gentian, Moss, Norway) on a Roche Modular P800 chemistry platform (Roche, Mannheim, Germany). The CysC test is linear from 0.41 mg/L to 8.0 mg/L. Overall CV 2.0% at a level of 0.96 mg/L and 1.0% at a level of 3.34 mg/L.

### Outcome variables

AKI was defined according to the KDIGO definitions [5]. pCr and hourly UO, using six hours sliding timeframe, were independently used to estimate KDIGO stage. In patients where both pCr and UO met KDIGO AKI criteria, the highest AKI score was applied. Reference pCr was based on ideal pCr, which was calculated assuming a creatinine clearance of 75 ml/min/1.73m^2^ using the ‘modification of diet in renal disease’ (MDRD) formula.

Diagnostic and prognostic accuracy was calculated using Receiver Operating Characteristic (ROC) curves. Diagnostic reference values were calculated using NGAL and CysC plasma levels on the day of AKI diagnosis in the AKI group, and biomarker plasma levels on the day of admission in the non-AKI group. Youdens index was then applied to identify the reference values which maximized sensitivity and specificity. The reference value with the highest Youdens index was chosen. For prognostic reference values the same procedure was applied; NGAL and CysC plasma values one day prior to AKI diagnosis were used in the AKI group, and NGAL and CysC plasma values on the day of admission in the non-AKI group.

### Statistical analysis

Data were analyzed using SPSS (SPSS Inc., version 26.0, Chicago, Illinois). Continuous variables were given as means with standard deviation (SD) in normal distribution or as medians with interquartile ranges (IQR) in non-normal distributions. Normality was tested using Kolmogorov-Smirnov and Shapiro-Wilk test, where *p* < 0.05 a non-normal distribution was assumed. Categorical variables were given as numbers and percentages. Ratios were compared using chi-squared test and means using Student’s T-test and for non-parametric values Mann-Whitney test or Kruskal-Wallis test was used.

## Results

**Fig. 2:**
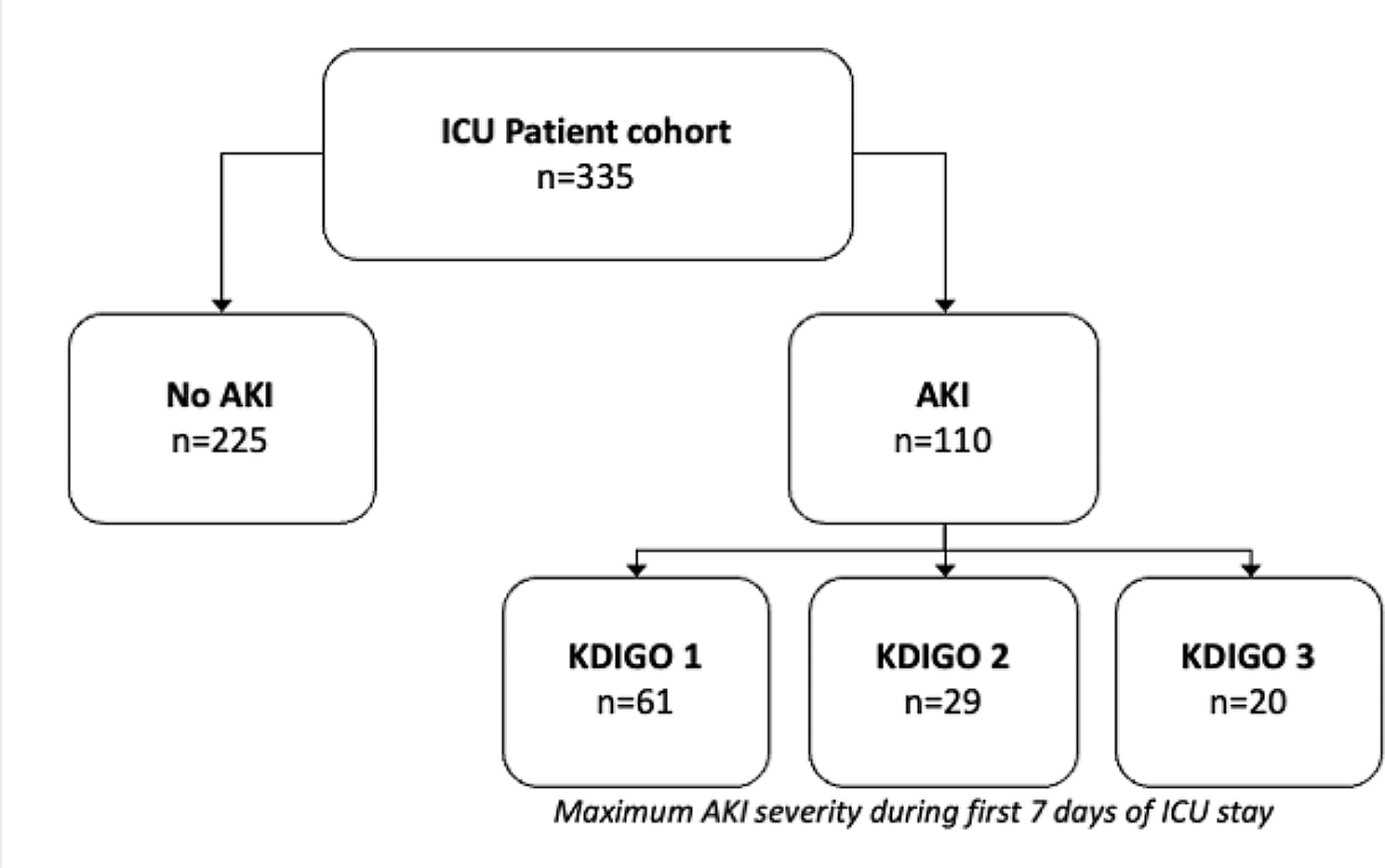
Acute Kidney Injury ICU cohort; AKI Acute Kidney Injury; KDIGO AKI Scores based on serum creatinine and urine output [5]

For this study we included a total of 335 patients; 110 patients met the KDIGO criteria for AKI at some point during the first seven days of ICU admission (Fig. 1, 2). Forty-nine patients showed new onset or progression of AKI during ICU stay. Twenty-five patients showed improvement of AKI or recovery or renal function during ICU stay (Supplemental Table 1).

### Patient characteristics

Both the AKI and non-AKI populations were mainly male (64.9% and 54.5% respectively *p* = 0.068) (Table 1). The AKI group was slightly older (62.6 years) than the non-AKI group (59.2 years) (*p* = 0.057). Patients with AKI had a significantly higher incidence of diabetes 23.6% vs. 13.8% (*p* = 0.024). Patients with AKI had significantly higher APACHE IV scores (*p* < 0.001). ICU mortality rate was not significantly different between both groups.

**Table 1:** Basic patient characteristics

	Non-AKI (*n* = 225)	AKI (*n* = 110)	*p*-value
**General Information**			
Age, mean (± SD)	59.2(± 15.3)	62.6 (± 15.4)	0.057
Male, n (%)	146 (64.9%)	60 (54.5%)	0.068
BMI mean (± SD)	25.8 (± 4.3)	27.6 (± 5.4)	< 0.001
**Comorbidities**, n (%)			
Diabetes	31 (13.8%)	26 (23.6%)	0.024
**Hospitalization**			
ICU Admission diagnosis, n (%)			
Elective Surgery	136 (60.4%)	36 (32.7%)	< 0.001
Emergency Surgery	26 (11.6%)	21 (19.1%)	
Acute Non-Surgical	63 (28.0%)	53 (48.2%)	
Sepsis/Infection	9 (4.0%)	13 (11.8%)	0.007
Hemorrhage	9 (4.0%)	7 (6.4%)	0.341
ICU admission days, median (IQR)*	1.0 (3.0)	2.5 (6.4)	< 0.001
**Clinical Severity**			
APACHE IV Score, median (IQR)	46.0 (29.0)	64.5 (54.0)	< 0.001
Mechanical Ventilation, n (%)	149 (66.2%)	90 (81.8%)	0.003
Mechanical Ventilation hours, median (IQR)*	9.0 (35.0)	20.0 (116.3)	< 0.001
RRT, n (%)	0 (0.0%)	14 (12.7%)	< 0.001
**Outcomes, n (%)**			
ICU Mortality	11 (4.9%)	10 (9.1%)	0.136

*Non-parametric Mann-Whitney based on Kolmogorov/Shaprio Wilk *p* < 0.05, AKI = Acute Kidney Injury, BMI = Body Mass Index, CKD = Chronic Kidney Disease, IQR = interquartile range, RRT = Renal Replacement Therapy,

### NGAL and CysC expression is higher in AKI patients, with higher biomarker levels in AKI patients with worse prognosis

**Fig. 3:**
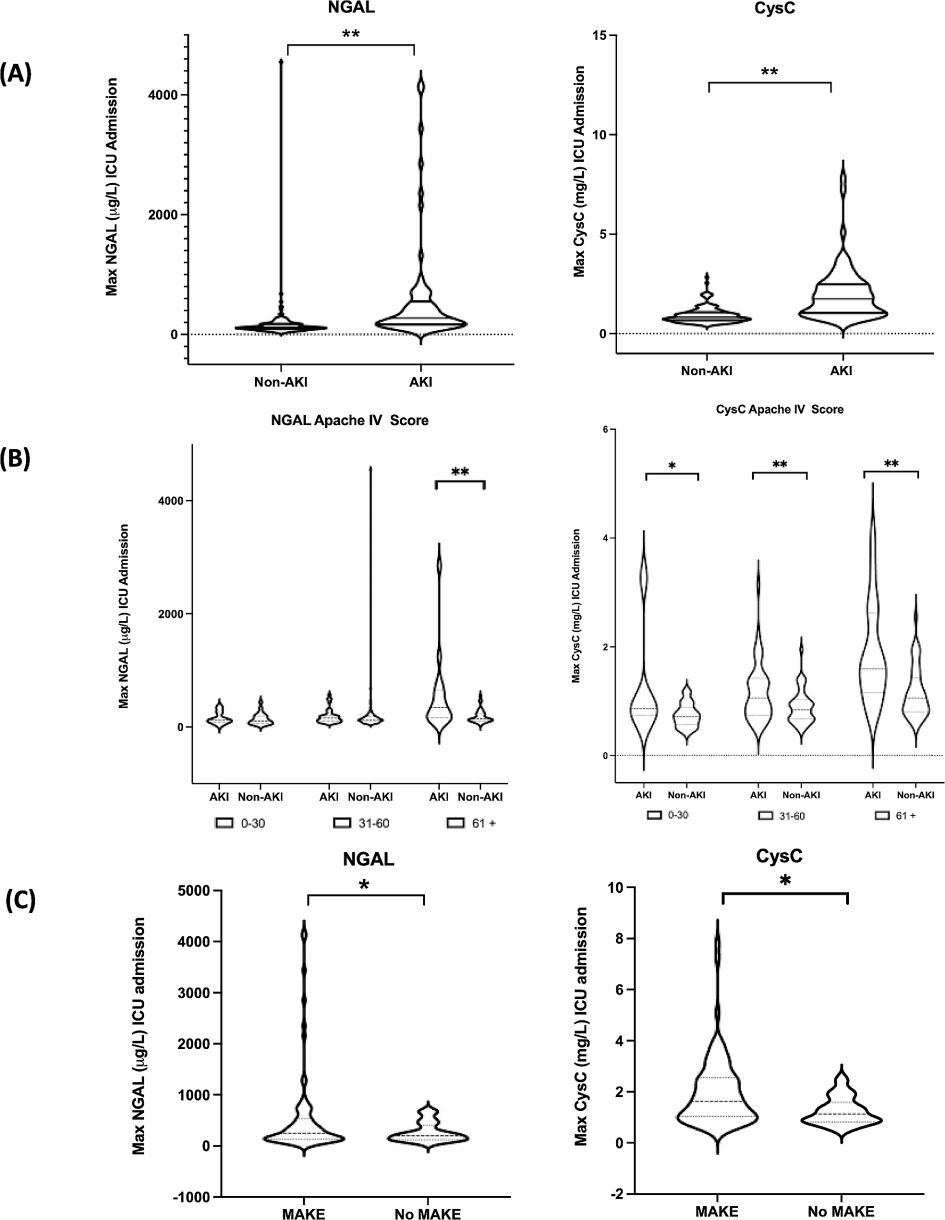
Maximum plasma NGAL and CysC during 7-day ICU admission. (**a**) AKI vs. non-AKI. (**b**) AKI vs. non-AKI based on APACHE IV score upon admission. (**c**) AKI only patients meeting Major Adverse Kidney Event (MAKE) outcomes. MAKE outcomes defined as AKI at ICU discharge, renal replacement therapy requirement during ICU admission or death during ICU admission

To study the relationship between plasma biomarker expression and AKI in our mixed ICU cohort, we began by looking at the maximum plasma NGAL and CysC expression during the 7-day ICU admission (Fig. 3). In our cohort we found a significantly higher plasma NGAL and CysC expression in AKI patients when compared to non-AKI (Fig. 3a). With the stratification of the population based on severity of critical illness based on APACHE IV score upon ICU admission we found that NGAL expression was only significantly higher in AKI patients with an APACHE IV score above 60 when compared to non-AKI (Fig. 3b). Plasma CysC expression was significantly higher in AKI patients compared to non-AKI in all APACHE IV groups. A comparison of all AKI patients based on the composite MAKE outcomes (AKI at ICU discharge, death, or RRT requirement) showed significantly higher NGAL and CysC expression in the MAKE group (Fig. 3c). Of note AKI vs. non-AKI as well as the MAKE vs. non-MAKE analyses show a large level of overlap in both NGAL and CysC levels raising the issue of the discriminative value of these biomarkers.

### NGAL and CysC show moderate diagnostic, and low prognostic accuracy for AKI in mixed ICU population

To determine the diagnostic and prognostic accuracy of plasma NGAL and CysC in our population we calculated the AUC on the day of diagnosis (diagnostic), and one day prior to AKI diagnosis (prognostic) (Supplemental Table 2). We found a NGAL diagnostic and prognostic AUC of 0.71 and 0.59, with a cut-off value of 234 µg/L and 118 µg/L respectively. CysC diagnostic and prognostic AUC was 0.75 and 0.59, with a cut-off value of 1.03 mg/L and 1.00 mg/L respectively. Of note, the day prior to AKI diagnosis, NGAL and CysC values were not available for KDIGO 3 patients.

### Plasma NGAL and CysC predict severity of AKI

**Fig. 4:**
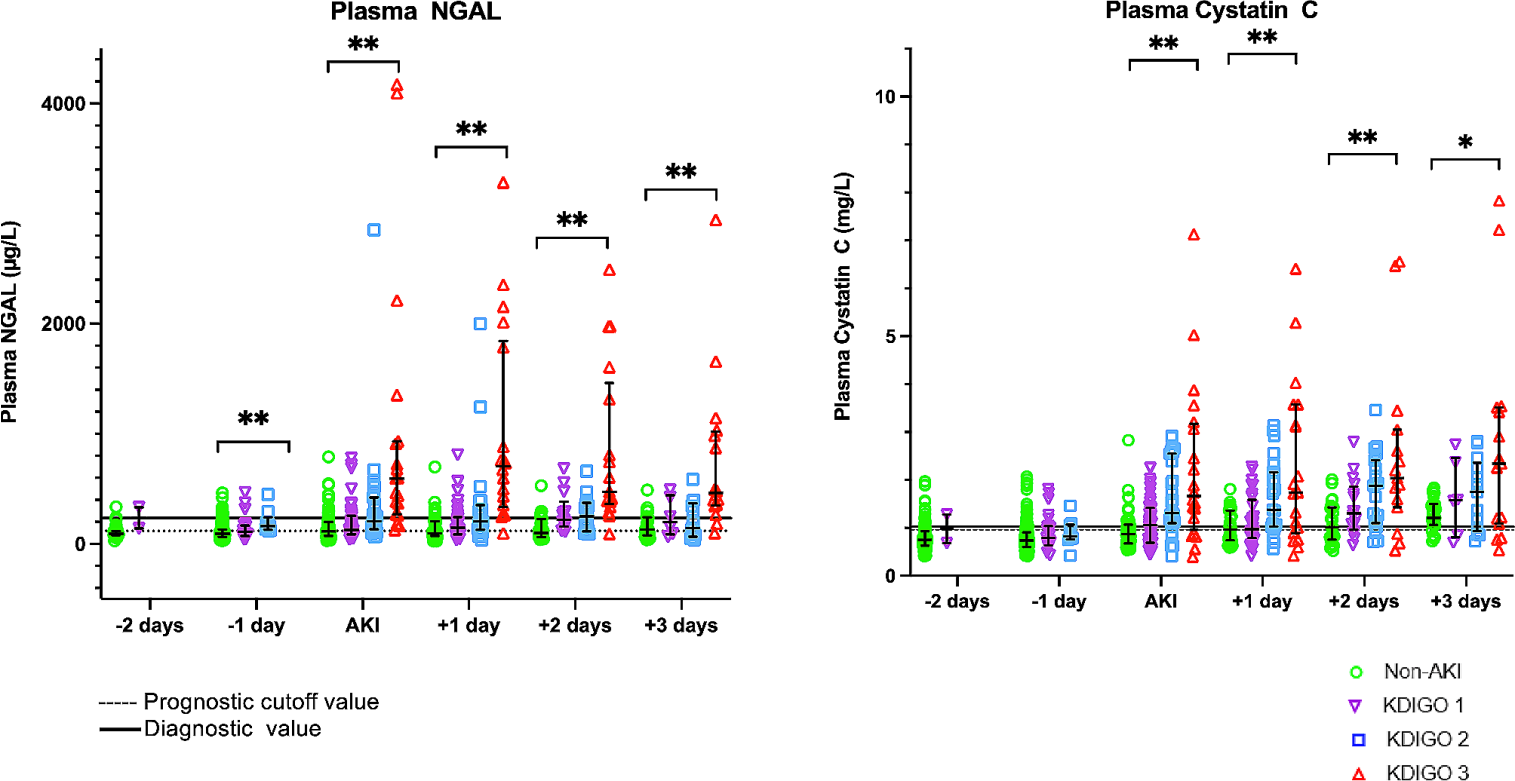
Plasma NGAL and Cystatin C during first 6 days of ICU admission based on maximum KDIGO score. KDIGO AKI score based on serum creatinine and urine output [5]. Individual biomarker plasma values, plus median and Interquartile Range (IQR) plotted. Prognostic and diagnostic cut off values calculated based on ROC and Youden’s principle. Prognostic cut off value based on biomarker levels 1 day prior to AKI diagnosis. Diagnostic cut off value based on biomarker levels on day of AKI diagnosis. ** *p* < 0.001, * *p* < 0.05

To better understand the clinical significance and evolution of plasma NGAL and CysC as biomarkers for AKI severity we studied the course of plasma NGAL and CysC based on the maximum KDIGO score during first six days of ICU stay. The higher KDIGO severity, the higher the plasma NGAL and CysC (Fig. 4). One day prior to AKI diagnosis, KDIGO 2 patients show median NGAL levels above the prognostic cut-off values. On the day of AKI diagnosis median NGAL levels were above the diagnostic cut-off values in KDIGO 3 patients. Only on day two after AKI diagnosis were the median NGAL levels above diagnostic value in KDIGO 2 patients. On the day of AKI diagnosis median CysC plasma levels were above diagnostic cut-off value for all AKI groups. Both biomarker levels remained elevated for three days after AKI diagnosis. The broad range of values is reflected by a wide IQR, which was highest amongst patients with KDIGO 3 AKI during follow up. These data imply that CysC and NGAL may accurately predict and diagnose severe AKI. However, the large distribution in individual values brings the clinical significance of these values into question.

### Various patterns of plasma NGAL and CysC expression in AKI

**Fig. 5:**
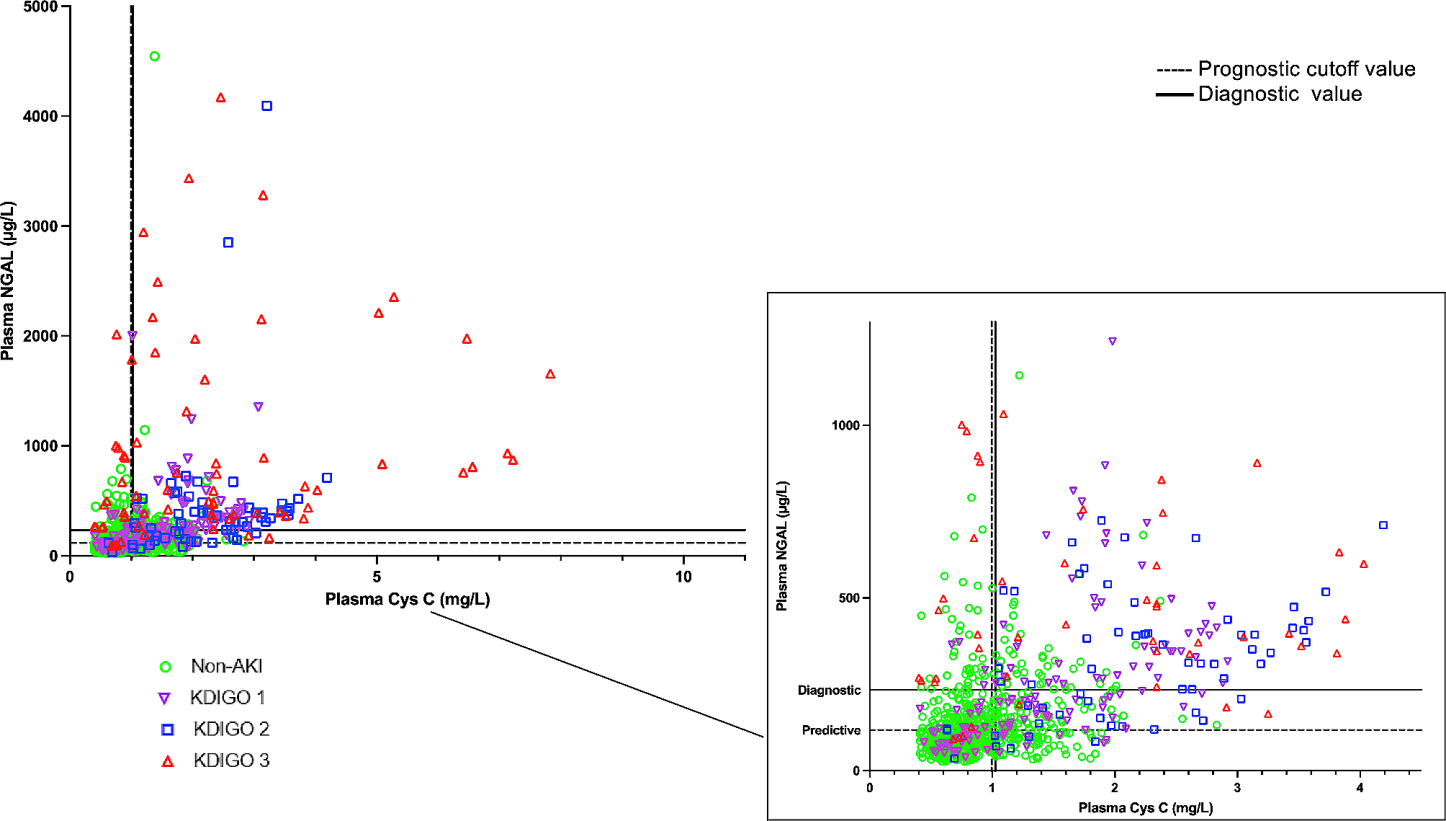
Plasma NGAL and Cystatin C scatter plot based on KDIGO score. KDIGO AKI score based on serum creatinine and urine output at time of biomarker measurement [5]. *Prognostic and diagnostic cut off values calculated based on ROC and Youden’s principle. Prognostic cut off value based on biomarker levels 1 day prior to AKI diagnosis. Diagnostic cut off value based on biomarker levels on day of AKI diagnosis*

**Fig. 6:**
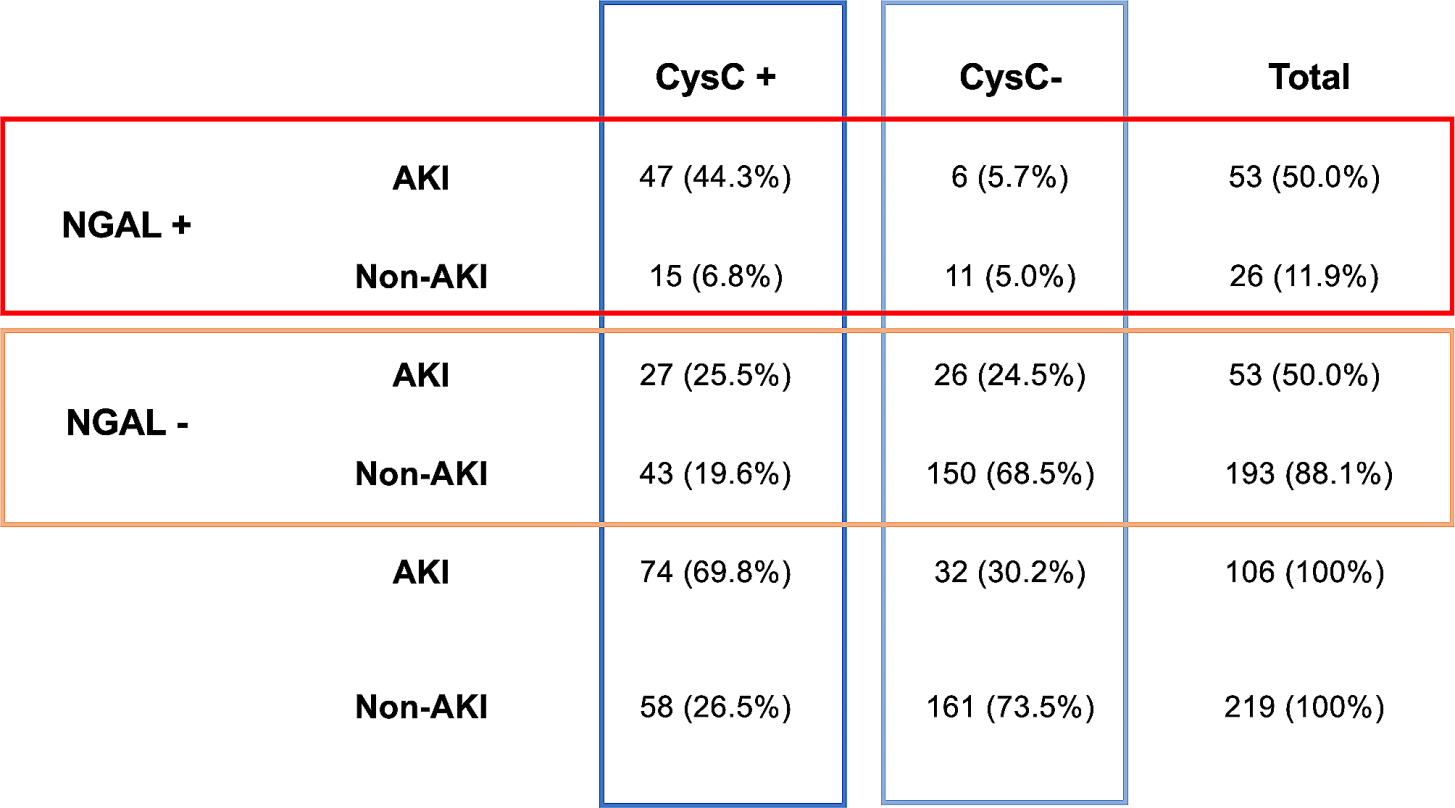
Classification of diagnostic biomarkers based on cut-off values. NGAL +: plasma NGAL above diagnostic cut-off (234 µg/L); NGAL -: plasma NGAL below diagnostic cut-off value. CysC +: plasma cystatin C above diagnostic cut-off value (1.03 mg/L). Cys -: plasma cystatin C below cut-off value

To further understand the distribution and relationship of plasma NGAL and CysC based on AKI severity we plotted individual biomarker values based on KDIGO AKI score (Fig. 5). We found no pattern in the main cluster. It is apparent that all KDIGO AKI groups are present in both elevated and non-elevated clusters of NGAL and CysC. This is further reflected in Fig. 6. In the AKI group NGAL and CysC are both above the diagnostic value in 44% of patients, while 25% of patients in this group have NGAL and CysC values below the diagnostic value. In the non-AKI group, 6.8% of patients have an NGAL and CysC levels above the diagnostic value, while in 69% both NGAL and CysC are below diagnostic values. This can also explain the wide IQR seen in Fig. 4. Together these figures show multiple patterns of plasma NGAL and CysC expression in AKI in a mixed ICU population.

## Discussion

The aim of this study was to investigate the timeline of plasma levels of NGAL and CysC and their relation to AKI severity during the first seven days of ICU admission in a mixed ICU population. Our data indicates that NGAL and CysC evolution over time is unpredictable. A high level of heterogeneity in biomarker expression poses a challenge for clinical application. Our data show a high variability in values with 56% of AKI patients having at least one biomarker *below* the diagnostic cut-off value, and 32% of non-AKI patients having at least one biomarker *above* the diagnostic cut-off value.

Our analysis of 110 AKI and 225 non-AKI patients shows higher plasma NGAL and CysC levels in AKI patients. Higher biomarker levels were also seen in AKI patients with MAKE outcomes. Within the AKI population there is a slight increase in NGAL and CysC levels one day prior to AKI. On the day of AKI diagnosis there is a sharp increase in both NGAL and CysC levels. A meta-analysis by Haase et al. [14] reports a diagnostic cut-off for plasma NGAL in critically ill patients of 155.0 µg/L with an AUC 0.728 and a prognostic cut-off for plasma NGAL of 179.2 (153.9-199.3) µg/L with an AUC 0.775 (0.679–0.869). Albert et al. [15] report a prognostic AUC of 0.773 (0.580–0.956) with a cut-off value of 165 µg/L. Compared to these meta-analyses, our data show a slightly higher diagnostic cut-off value with a comparable AUC (234 µg/L, AUC 0.71), while our prognostic cut-off value and AUC are lower (118 µg/L, AUC 0.59). Our lower prognostic AUC for NGAL may be partly explained by the population used by Haase et al. [14], although both populations are heterogenous, the distribution of each subpopulation may differ. In a review by Hjortrup et al. [16], authors report prognostic NGAL AUCs of 0.54 to 0.98 for the prediction of AKI in general ICU patients. These values are comparable to our results and may reflect a high level of heterogeneity within this population. Our prognostic CysC cut-off values are comparable to those reported by Yong et al. [17], however our AUC values are lower. Their prognostic cut-off values of plasma CysC in ICU populations were 0.80-1.54 mg/L with an AUC of 0.86 for a pooled diagnostic accuracy of ICU/ AKI. This again may reflect a high level of heterogeneity in our population. Biological variation may also play a role, with a reported CysC within subject variation of 3.9 (CI 3.9–8.1%) and between subject variation of 12.1 (CI 12.1-15.15%) [18]. This variation within and between populations makes the application of plasma NGAL and CysC difficult.

The underlying renal damage etiology is heterogeneous and is strongly influenced by comorbidities and clinical progression. These factors result in a disparity in plasma renal biomarker levels. Different biomarker profiles may reflect disease sub-phenotypes and different renal areas of damage and/or repair. Functional biomarkers such as pCr and CysC give an indication of glomerular filtration, and NGAL and KIM-1 reflect tubular damage [2, 3]. In two separate studies higher plasma NGAL expression was seen in sepsis-AKI compared to non-sepsis AKI [8, 19]. Also, in their meta-analysis Haase et al. [14] show different NGAL cut-off values depending on patient setting. However, in a previous study we showed differences in NGAL and kidney injury molecule (KIM)-1 protein expression in post-mortem sepsis-AKI renal biopsies, suggesting that etiology alone may not account for all differences [7]. Inflammatory status and co-morbidities may also play a role in biomarker expression. Due to our small sample size we were not able to complete a subgroup analysis based on comorbidities to further study this. Both NGAL and CysC are not only expressed in the kidney and therefore systemic changes during critical illness may influence circulating levels [3, 20]. In our data we show that NGAL expression was only significantly higher in AKI patients with an APACHE IV score above 60 when compared to non-AKI, while CysC levels were significantly higher regardless of critical illness score. This suggests that NGAL may underestimate AKI in less critically ill patients, while CysC appears to be able to differentiate between AKI and non-AKI regardless of critical illness score. Lastly, timing may play a role. Different plasma biomarkers have different peak times. Unfortunately, there are no clinical signs which may help identify an impending renal event. This highlights the necessity for biomarkers as a clinical tool. Considering the current data and those published by others, we would argue for the clinical use of a biomarker panel which may aid clinical reasoning regarding pathophysiology and perhaps help identify treatment targets.

Another possible explanation for the wide range of cut-off values and AUCs may be the AKI definition itself. The current gold standard to define AKI is elevated pCr with or without decreased UO [21, 22]. Both UO and pCr have acknowledged confounding factors and limitations. Additionally, these criteria are strongly influenced by the way in which AKI definition is applied [1, 23]. Therefore, renal biomarkers are tested against a flawed AKI definition. This has led to the introduction of the term ‘subclinical AKI’; patients who do not meet the current criteria for AKI but present with elevated circulating biomarker levels [22, 24]. This subclinical AKI may reflect limited tubular damage and/or an innate compensation of glomerular filtration by non-injured glomeruli (renal functional reserve). It has been suggested that early intervention in this group of patients may prevent further progression and thereby may improve clinical outcomes. Based on this, the Acute Dialysis Quality Initiative (2013) [25] recommended the use of biomarkers to complement the traditional AKI criteria. Since then, various studies have evaluated the use of biomarkers in identifying ‘high risk’ patients with subclinical AKI and consequently early implementation of components of the KDIGO guidelines in these patients. These studies report a reduced incidence of moderate to severe AKI in well-defined, homogeneous, patient groups with a common AKI etiology such as post-cardiac surgery and post-abdominal surgery [11, 26]. This suggests that renal biomarkers may have a role in identifying high risk patients in pre-selected groups. No consistent findings have yet been found in acute patients.

### Limitations

In this study we provide a longitudinal analysis of plasma NGAL and CysC in a mixed ICU population. The limitations are as follows. The relatively small sample size with a high level of patient heterogeneity results in multiple patient subgroups. We acknowledge that further sensitivity analyses in specific patient categories such as diabetes and sepsis would be of interest, however due to the small sample size we were unable to properly investigate this. In our study we used the KDIGO definition using both pCr and UO since this is the gold standard for current detection of AKI. The fact that we used an estimated pCr, assuming normal kidney function, rather than a known baseline pCr, possibly underestimates our AKI incidence [27, 28]. All patients with known chronic kidney disease (CKD) were removed from analyses to limit false positive results. Lastly, not all patients completed a full seven-day ICU admission limiting our biomarker data over time.

### Future perspectives

With our study we aimed to study two AKI biomarkers in a heterogeneous critical care unit as to simulate the normal clinical setting. To apply the renal biomarkers in routine critical care, we believe that the use of two or more biomarkers is required. Future research should focus on identifying these subgroups which may relate to predisposing factors, triggers for AKI and differences in pathophysiological mechanisms. The costs associated with biomarker analyses remains the biggest obstacle for the application of multiple renal biomarkers in de clinics and in research.

## Conclusion

NGAL and CysC as biomarkers for the early detection of AKI may besubject to both pathophysiological, as well as patient related heterogeneity. This poses a challenge in the clinical application of these biomarkers. Further understanding of individual biomarker profiles with their relation to pathophysiology and timing may help in their application in mixed populations, and a deeper understanding of AKI as a heterogeneous disease with different disease sub-phenotypes.

### Electronic supplementary material

Below is the link to the electronic supplementary material.

Supplementary Material 1

## Data Availability

The datasets used and/or analyzed during the current study are available from the corresponding author on reasonable request.

## Abbreviations

AKI: Acute Kidney Injury
pCr: Plasma creatinine
UO: Urine output
MAKE: Major Adverse Kidney Events
RRT: Renal replacement therapy
NGAL: Neutrophil gelatinase-associated lipocalin
CysC: Cystatin C
ICU: Intensive Care Unit
CKD: Chronic kidney disease
PDMS: Patient data management system
CV: Coefficient of variation
PETIA: Particle enhanced turbidimetric immunoassay
MDRD: Modification of disease in renal disease
ROC: Receiver operating characteristic
SD: Standard deviation
IQR: Interquartile ranges
