# Supplementary material for: Longitudinal NGAL and cystatin C plasma profiles present a high level of heterogeneity in a mixed ICU population
Source: BMC Nephrol. 2024 Jan 29;25:43. doi: 10.1186/s12882-024-03477-2 (PMC10826252; doi:10.1186/s12882-024-03477-2)
Supplement: Supplementary file 1 — Supplementary Material 1 [file 12882_2024_3477_MOESM1_ESM.docx]

**Supplementary Data**

# 1.0 Individual evolution of renal biomarker expression over time

Consequently, we looked at the individual plasma NGAL and CysC progression during the first 6 days of ICU admission. Individually plotted renal biomarker levels show no pattern of evolution over a 6-day timeline **Supplemental Fig 2**. As expected, the AKI group appears to have higher NGAL and CysC levels when compared to the non-AKI group. However, in the low biomarker group, there is a high level of overlap amongst the AKI and non-AKI group. In some patients the biomarker value is at its highest on the day of AKI diagnosis, while in others the biomarker value increases after AKI diagnosis. In the non-AKI group, fluctuations in the biomarker values are also present throughout the 6-day timeline. These results show a high level of individual variation over time in plasma NGAL and CysC expression in AKI patients in a mixed ICU.

# 2.0 Supplementary Tables and Figures

**
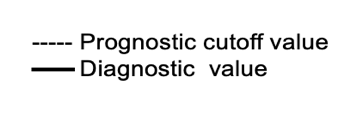
**


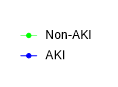


**Supplemental Fig 1:** Individual plasma NGAL and Cystatin C levels based on KDIGO score. KDIGO AKI score based on serum creatinine and urine output at time of biomarker measurement (5). Prognostic and diagnostic cut off values calculated based on ROC and Youden’s principle. Prognostic cut off value based on biomarker levels 1 day prior to AKI diagnosis. Diagnostic cut off value based on biomarker levels on day of AKI diagnosis.

| **Supplementary Table 1:** Evolution of AKI severity during first 7 days ICU admission. | | | | |
| --- | --- | --- | --- | --- |
|  | Final KDIGO score during ICU admission | | | |
| Admission KDIGO-score | KDIGO 0 | KDIGO 1 | KDIGO 2 | KDIGO 3 |
| KDIGO 0 | 225 | 25 | 10 | 0 |
| KDIGO 1 | 16 | 20 | 5 | 5 |
| KDIGO 2 | 1 | 6 | 7 | 4 |
| KDIGO 3 | 2 | 0 | 0 | 9 |
| *KDIGO AKI Scores based on serum creatinine and urine output* (5). | | | | |

| **Supplemental Table 2:** Plasma NGAL and Cystatin C Diagnostic and Prognostic Cut-off Values | | | | | | |
| --- | --- | --- | --- | --- | --- | --- |
| Biomarker | | AUC | Cut-off value | Youdens Index | Specificity | Sensitivity |
| NGAL | Diagnostic | 0.71 | 234 µg/L | 35.8 | 92.5 | 43.3 |
|  | Prognostic | 0.59 | 118 µg/L | 22.5 | 60.0 | 62.5 |
| Cystatin C | Diagnostic | 0.75 | 1.03 mg/L | 47.6 | 82.5 | 65.1 |
|  | Prognostic | 0.59 | 1.00 mg/L | 19.4 | 81.9 | 37.5 |

| **Supplemental Table 3**: Overview missing data | | | | | | | | |
| --- | --- | --- | --- | --- | --- | --- | --- | --- |
| Follow-up days | 1 | 2 | 3 | 4 | 5 | 6 | 7 | total |
| First day inclusion |  |  |  |  |  |  |  |  |
| 1 | 20 | 151 | 34 | 12 | 10 | 9 | 27 | 263 |
| 2 | 26 | 7 | 3 | 1 | 1 | 5 | 0 | 43 |
| 3 | 7 | 4 | 2 | 4 | 3 | 0 | 0 | 20 |
| 4 | 1 | 0 | 0 | 0 | 0 | 0 | 0 | 1 |
| 5 | 1 | 2 | 1 | 0 | 0 | 0 | 0 | 4 |
| 6 | 1 | 3 | 0 | 0 | 0 | 0 | 0 | 4 |
| 7 | 0 | 0 | 0 | 0 | 0 | 0 | 0 | 0 |
| total | 56 | 167 | 40 | 17 | 14 | 14 | 27 |  |
| Missing Data | | | | | | | | |
| *EXPECTED* | 263 | 287 | 131 | 82 | 66 | 56 | 39 |  |
| NGAL | 21 | 9 | 12 | 5 | 4 | 3 | 0 | 54 |
| CysC | 20 | 8 | 6 | 2 | 5 | 1 | 0 | 43 |
